# Selective antibody activation through protease-activated pro-antibodies that mask binding sites with inhibitory domains

**DOI:** 10.1038/s41598-017-11886-7

**Published:** 2017-09-14

**Authors:** I-Ju Chen, Chih-Hung Chuang, Yuan-Chin Hsieh, Yun-Chi Lu, Wen-Wei Lin, Chien-Chiao Huang, Ta-Chun Cheng, Yi-An Cheng, Kai-Wen Cheng, Yeng-Tseng Wang, Fang-Ming Chen, Tian-Lu Cheng, Shey-Cherng Tzou

**Affiliations:** 1 Graduate Institute of Medicine, Kaohsiung Medical University, Kaohsiung, Taiwan; 2 Department of Medical Laboratory Science and Biotechnology, College of Health Sciences, Kaohsiung Medical University, Kaohsiung, Taiwan; 3 Center for Biomarkers and Biotech Drugs, Kaohsiung Medical University, Kaohsiung, Taiwan; 4 Institute of Biomedical Sciences, National Sun Yat-Sen University, Kaohsiung, Taiwan; 5 Graduate Institute of Clinical Medicine, Kaohsiung Medical University, Kaohsiung, Taiwan; 6 Department of Biomedical Science and Environmental Biology, Kaohsiung Medical University, Kaohsiung, Taiwan; 7 Department of Biochemistry, Kaohsiung Medical University, Kaohsiung, Taiwan; 8 Department of Surgery, Faculty of Medicine, College of Medicine, Kaohsiung Medical University, Kaohsiung, Taiwan; 9 Department of Medical Research, Kaohsiung Medical University Hospital, Kaohsiung, Taiwan; 10 Institute of Molecular Medicine and Bioengineering, Department of Biological Science and Technology, National Chiao Tung University, Hsin-Chu, Taiwan

## Abstract

Systemic injection of therapeutic antibodies may cause serious adverse effects due to on-target toxicity to the antigens expressed in normal tissues. To improve the targeting selectivity to the region of disease sites, we developed protease-activated pro-antibodies by masking the binding sites of antibodies with inhibitory domains that can be removed by proteases that are highly expressed at the disease sites. The latency-associated peptide (LAP), C2b or CBa of complement factor 2/B were linked, through a substrate peptide of matrix metalloproteinase-2 (MMP-2), to an anti-epidermal growth factor receptor (EGFR) antibody and an anti-tumor necrosis factor-α (TNF-α) antibody. Results showed that all the inhibitory domains could be removed by MMP-2 to restore the binding activities of the antibodies. LAP substantially reduced (53.8%) the binding activity of the anti-EGFR antibody on EGFR-expressing cells, whereas C2b and CBa were ineffective (21% and 9.3% reduction, respectively). Similarly, LAP also blocked 53.9% of the binding activity of the anti-TNF-α antibody. Finally, molecular dynamic simulation showed that the masking efficiency of LAP, C2b and CBa was 33.7%, 10.3% and −5.4%, respectively, over the binding sites of the antibodies. This strategy may aid in designing new protease-activated pro-antibodies that attain high therapeutic potency yet reduced systemic on-target toxicity.

## Introduction

Many monoclonal antibodies have been approved for treating malignant cancers^1–3^, inflammatory diseases such as rheumatoid arthritis^4, 5^ and osteoporosis^6^. According to a recent survey, about 50 monoclonal antibodies are currently approved for clinical use and approximately 350 monoclonal antibodies are under development^7^. Unlike many small molecule drugs that may target multiple proteins, monoclonal antibodies typically target specific molecules (antigens) associated with diseases. Conceptually, monoclonal antibodies should display higher specificity and have fewer systemic side effects than small molecule drugs.

In some cases, however, the antigens targeted by therapeutic antibodies are not expressed exclusively at the disease sites. Systemic injection of therapeutic monoclonal antibodies may cause considerable adverse effects^8^ and thus decrease treatment efficacies. For example, epidermal growth factor receptor (EGFR) is over-expressed in some tumor cells and plays an important role in tumor progression^9^. However, EGFR is also expressed in some epithelial cells^10, 11^. Systemic injection of anti-EGFR antibodies (Erbitux) is known to induce adverse effects such as skin rash in 80–90% of patients with metastatic colorectal cancer^12^. Likewise, tumor necrosis factor-α (TNF-α) is a pro-inflammatory cytokine over-expressed in the joints of rheumatoid arthritis (RA) patients^13^, but TNF-α is also an important cytokine for defense against microbial infections^14–16^. Systemic targeting of TNF-α with anti-TNF-α antibodies (Remicade^17^ and Humira^18^) is known to bear higher risk of serious infections (odds ratio: 2.01)^19, 20^, and long-term treatment with Remicade may increase lymphoma incidence^19, 21^. To overcome these on-target toxicities and reduce the related adverse effects, we engineered a pro-antibody that can be selectively activated in the region of disease sites. Specific localization of the therapeutic antibody may lower on-target toxicities of the antibodies.

**Figure 1:**
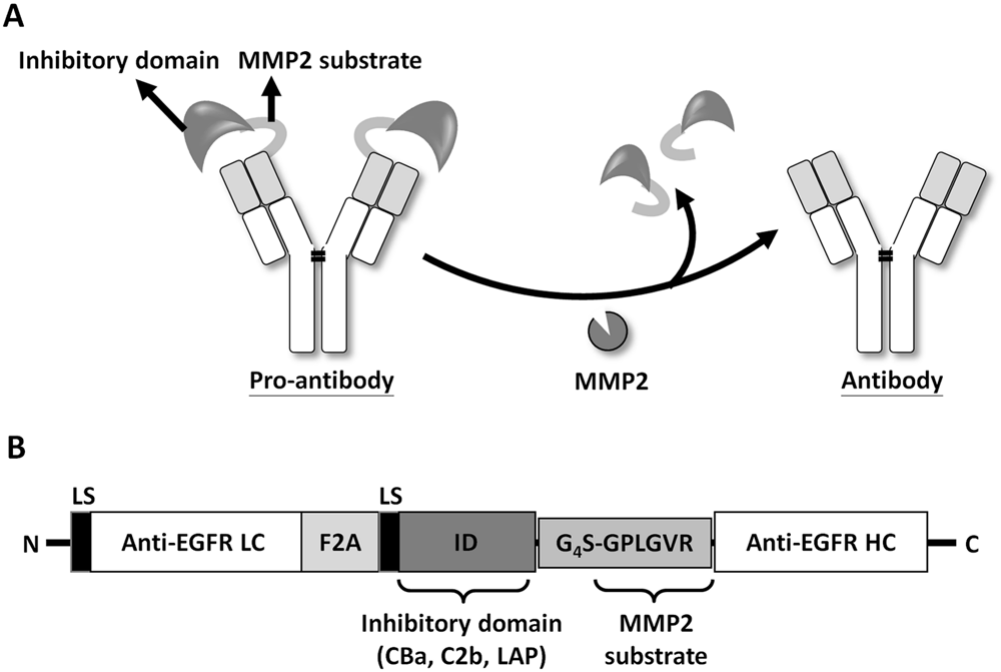
Development of the protease-activated pro-antibodies via an inhibitory domain for selective antibody activation. (**A**) Schematic representation of the design and activation of the pro-antibody by MMP-2 overexpressed at the disease sites. The inhibitory domains sterically block the binding sites of the antibody and render the antibody inactive unless they are removed by MMP-2. (**B**) The pro-antibody gene encodes a Igκ leader sequence (LS), the anti-EGFR antibody light chain (LC), a furin-2A bicistronic element sequence (F2A), the inhibitory domain (ID) including CBa of CB, C2b of C2 or LAP of TGF-β, the G_4_S flexible linker (GGGGS), the MMP-2 substrate sequence (GPLGVR) and anti-EGFR antibody heavy chain (HC).

In this study, we developed protease-activated pro-antibodies to direct antibody action solely to disease sites. This pro-antibody strategy involves masking the antibody binding sites by inhibitory domains derived from latency-associated peptide (LAP) of transforming growth factor-β (TGF-β) and C2b of complement factor 2 and CBa of complement factor B, through a substrate peptide (GPLGVR) for matrix metalloproteinase-2 (MMP-2)^22^, to the heavy chain of the antibody. The inhibitory domains were expected to block the binding activity of the pro-antibody until MMP-2 activation (Fig. 1). We selected the inhibitory domains based on two principles. First, the sequences must come from endogenous proteins such that the probability of provoking anti-inhibitory domain immune responses is minimized. Second, the inhibitory domains must not display apparent or known biological function (such as enzyme) other than blocking the activity of the original proteins. According to current knowledge, the function of complement factor Ba (CBa) were unknown except the small fragment of CB^23^. The function of C2b were the precursor of vasoactive C2 kinin, but still disputed^24^. The LAP is the propeptide of TGF-β which can localize latent TGF-β to the latent-TGF-β-binding protein (LTBP) to help TGF-β activation^25^. Once cleaved from the latent TGF-β, no obvious activity is attributed to LAP. We constructed and produced CBa-, C2b-, and LAP-masked anti-EGFR antibodies and a LAP-masked anti-TNF-α antibody. The binding activities of the pro-antibodies were tested and compared in the presence or absence of MMP-2 *in vitro*. Our results indicate that masking antibody binding sites may be an effective way to prevent or reduce adverse effects during monoclonal antibody therapy by allowing antibody binding to antigens at disease sites (protease positive) but not in normal tissues (protease negative).

## Results

### Construction of inhibitory domain-masked pro-anti-EGFR antibodies

To test whether inhibitory domains can mask binding of antibodies, we surveyed several proteins in which cleavage of pro-domains is necessary to activate the proteins, including factor 2 (C2b of C2) and factor B (CBa of CB) of the complement system, and the transforming growth factor-β (LAP of TGF-β). These pro-domains were selected as inhibitory domain because they are derived from endogenous proteins and do not retain apparent biological function (such as enzyme) other than blocking the activity of the original proteins. The inhibitory domains were linked to the N-terminus of heavy chain of an anti-EGFR antibody through a substrate peptide of MMP 2 by DNA cloning, as illustrated in Fig. 1. The light chain and the engineered heavy chains were then linked through a furin 2A cleavage sequence and cloned to retroviral vector pLHCX to form C2b-anti-EGFR, CBa-anti-EGFR, and LAP-anti-EGFR expression vectors.

### Removal of inhibitory domains from pro-anti-EGFR Abs by MMP-2

**Figure 2:**
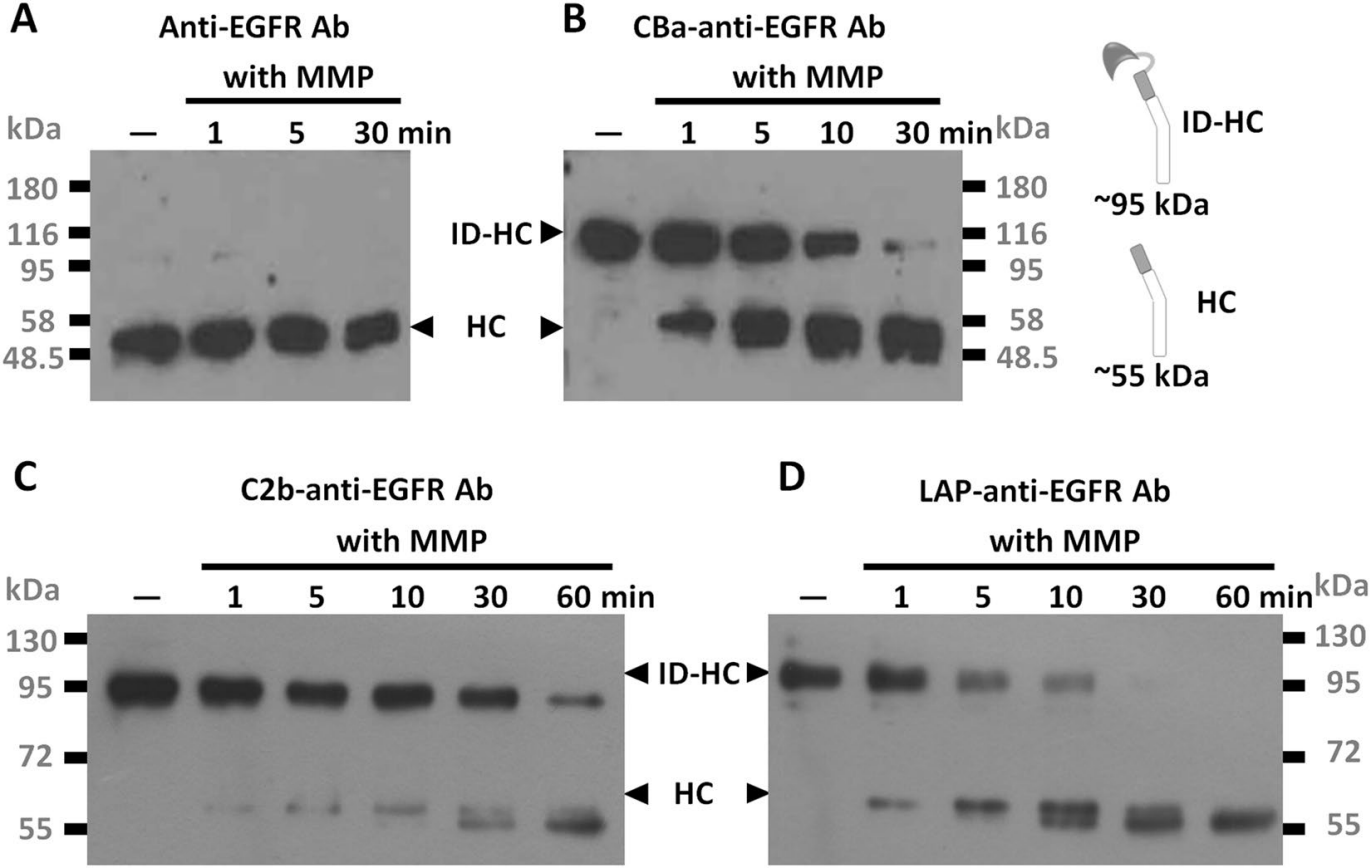
Removal of inhibitory domains from recombinant pro-antibodies by MMP-2. Unmodified anti-EGFR antibody (**A**), CBa-anti-EGFR antibody (**B**), C2b-anti-EGFR antibody (**C**) or LAP-anti-EGFR antibody (**D**) were digested by MMP-2 for the indicated time. Digested antibodies were resolved by reducing SDS-PAGE and analyzed by western blot using HRP-conjugated goat anti human-IgG Fcγ antibodies. Note that amounts of cut (mature form) antibodies increased as digestion was prolonged from 1 minute to 60 minutes. ID-HC: inhibitory domain-heavy chain. HC: heavy chain. with MMP: with MMP-2 incubation. The original blots image for panel A–D are presented in Supplementary Figure S5.

To test whether the inhibitory domains can be cleaved from the heavy chains of the recombinant antibodies by MMP-2, we incubated culture supernatants from 293T transfectants that expressed CBa-anti-EGFR antibody, C2b-anti-EGFR antibody and LAP-anti-EGFR antibody with MMP-2 for different amounts of time. Initially, the pro-antibody predominated; however, as reaction time increased the cleaved (mature) antibodies, as indicated by smaller bands in western blots, successively increased (Fig. 2B–D). Cleaved antibodies migrated similarly as the native (unmodified) anti-EGFR antibodies in the reducing SDS-PAGE, indicating MMP-2 cleaved at the predicted position within the substrate peptide of the recombinant antibodies. These data suggested the inhibitory domains can be effectively removed by disease-associated proteases (MMP-2). As a control, unmodified anti-EGFR antibody was not digested by the MMP-2 in all the conditions tested (Fig. 2A). Size-exclusion high-performance liquid chromatography analyses showed similar results between anti-EGFR antibodies and LAP-anti-EGFR antibodies, with a major peak detected in both HPLC and Coomassie staining, indicating monomeric conformation. A minor peak preceding the major peak may indicate slight aggregation in both of anti-EGFR and LAP-anti-EGFR antibodies in solution, but the aggregation of LAP-anti-EGFR antibodies were not more severe than anti-EGFR antibodies, indicating the LAP domain does not cause aggregation of pro-antibodies to a significant extent. (supplementary Figure 1). We further confirmed the clipped inhibitory domains dissociate from anti-EGFR antibodies by immunoprecipitation in supplementary Figure 2, which shows that LAP domain (with a molecular weight approximately 40 kDa) was not co-precipitated (thus not seen in the gel) with the antibodies treated by MMP-2. This data support the idea that clipped inhibitory domains would likely diffuse away from the anti-EGFR antibodies after digestion.

### Recovery of binding activities of the pro-anti-EGFR Abs after MMP-2 digestion

**Figure 3:**
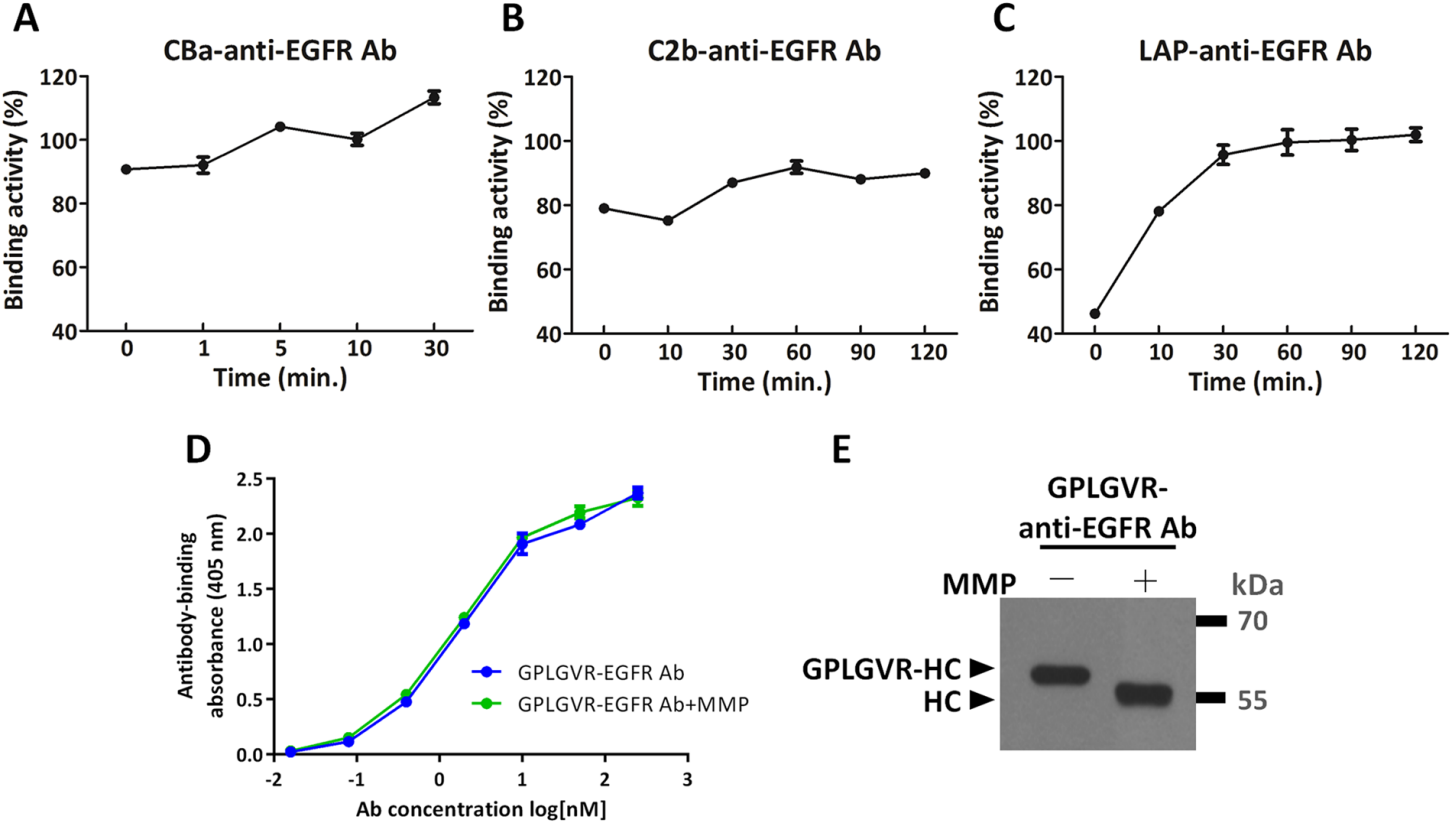
Recovery of binding activities of the recombinant antibodies after removal of inhibitory domains by MMP-2. CBa-anti-EGFR antibody (**A**), C2b-anti-EGFR antibody (**B**) or LAP-anti-EGFR antibody (**C**) that were digested by MMP-2 for indicated time were added to microtiter plates pre-coated with MDA-MB-468 cells. Degree of antibody binding to the cells was compared to the binding of the cells by the unmodified anti-EGFR antibody (set as 100%). The LAP domain reduced antibody binding activity by 53.8%. The CBa and C2b domains masked antibody binding sites ineffectively, only marginally reducing the antibody binding activity by 9.3% and 21%, respectively. Note that after MMP-2 digestion of the LAP-anti-EGFR antibody, the binding activity rose progressively from 46.2% to 100%. (**D**) The binding activities of MMP-2 substrate peptide-linked anti-EGFR antibody (GPLGVR-anti-EGFR Ab) and MMP-2 substrate peptide-removed anti-EGFR antibody (GPLGVR-anti-EGFR Ab + MMP) showed the MMP-2 substrate peptide does not affect the binding activity of the antibody. (**E**) MMP-2 digested antibodies were resolved by reducing SDS-PAGE and analyzed by western blot using HRP-conjugated goat anti human-IgG Fcγ antibodies. GPLGVR peptide was indeed removed from the heavy chains of anti-EGFR antibodies by MMP-2, as indicated by reduction of molecular size. GPLGVR-HC: MMP-2 substrate peptide-linked heavy chain. HC: heavy chain. The original blot image for panel E is presented in Supplementary Figure S6.

We next tested whether the binding activities of the recombinant antibodies were inhibited in the presence of the inhibitory domains and whether MMP-2 digestion was capable of restoring the activities of the antibodies. The culture supernatants from cells expressing CBa-anti-EGFR, C2b-anti-EGFR or LAP-anti-EGFR antibodies were digested by MMP-2 for different amounts of time and then tested by a cell-based ELISA where EGFR-expressing MDA-MB-468 cells were used as an antigen source. Prior to MMP-2 digestion, CBa, C2b and LAP domains reduced 9.3%, 21% and 53.8%, respectively, binding activities of the recombinant antibodies when compared to binding activity of the unmodified anti-EGFR antibodies. Removal of the inhibitory domains restored binding activities of the antibodies (Fig. 3). In general, longer incubation with MMP-2 better recovered antibody binding activity, in line with more abundant mature antibodies seen in western blot analyses in Fig. 2. CBa (Fig. 3A) and C2b (Fig. 3B) masked antibody binding sites ineffectively, enhancement of antibody binding after MMP-2 treatment was not as evident as LAP-anti-EGFR antibody, which rose progressively from 46.2% to 100% after treatment with MMP-2 for 1 hour (Fig. 3C). These data suggest that LAP is most effective in masking the binding sites of antibodies and that masking can be fully removed by MMP-2. To confirm the masking effect of pro-antibodies is dependent on inhibitory domains, rather than the MMP-2 substrate peptide (GPLGVR), we produced and purified recombinant MMP-2 substrate peptide-linked anti-EGFR antibodies (GPLGVR-anti-EGFR Abs). The binding activity of GPLGVR-anti-EGFR antibodies was indistinguishable with or without MMP-2 treatments (Fig. 3D). We also confirmed that the GPLGVR peptide was successfully removed from the heavy chains of anti-EGFR antibodies by MMP-2 (Fig. 3E). Thus, masking of the binding activity of pro-antibodies is dependent on the inhibitory domain (LAP), not the MMP-2 substrate peptide (GPLGVR).

### The EC_50_ and binding specificities of LAP-anti-EGFR antibodies before and after MMP-2 digestion

**Figure 4:**
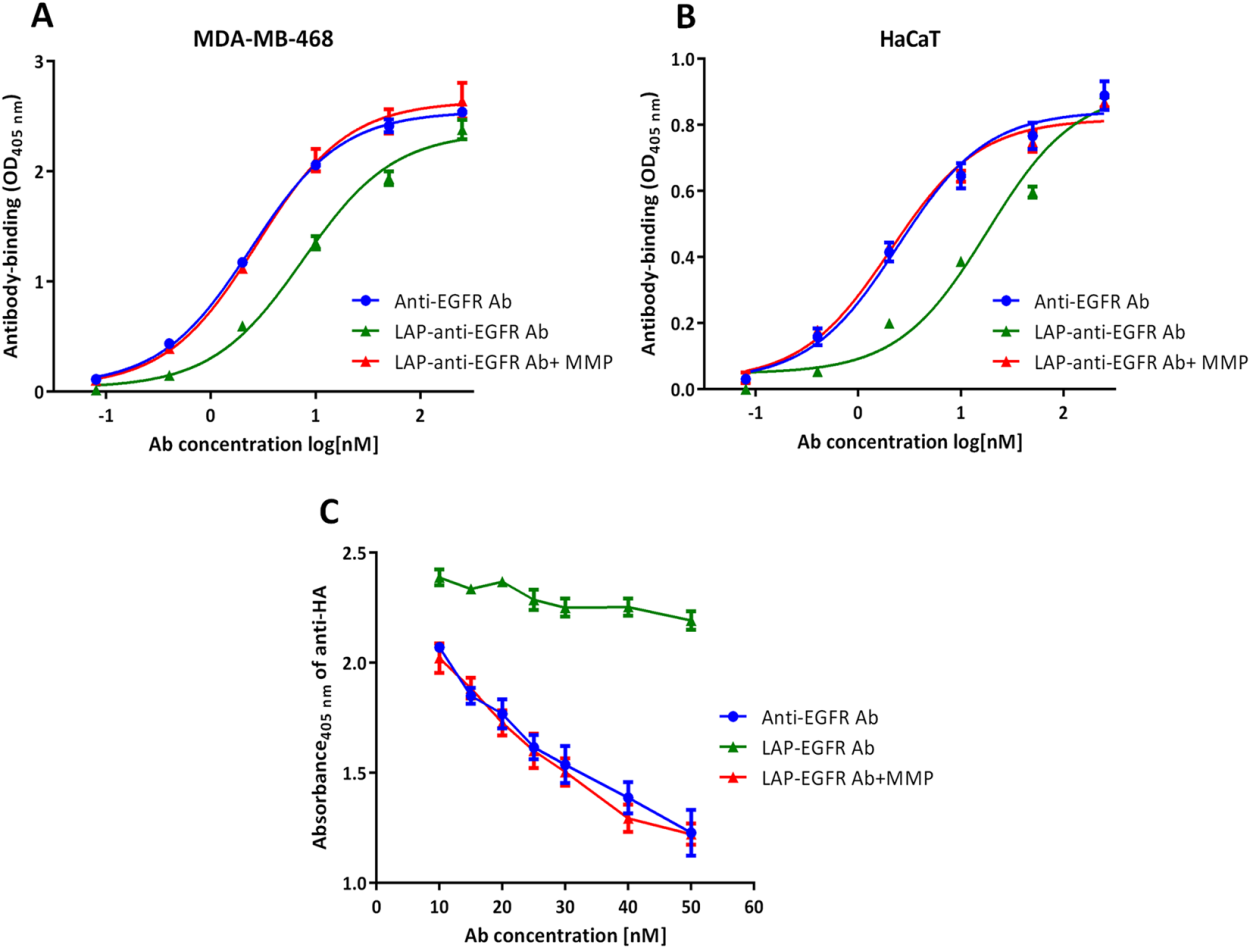
The EC_50_ and binding specificities of LAP-anti-EGFR antibodies before and after MMP-2 digestion. The binding kinetic of the anti-EGFR antibody (), LAP-anti-EGFR antibody () and MMP-2-activated LAP-anti-EGFR antibody () in (**A**) MDA-MB-468 and (**B**) HaCaT cells. Note that the curve of LAP-anti-EGFR antibody is shifted to the right in both groups. In the MDA-MB-468 cells (high EGFR expression), the EC_50_ values for the anti-EGFR antibody, LAP-anti-EGFR antibody and MMP-2-activated LAP-anti-EGFR antibody were 2.4 nM, 7.5 nM and 2.7 nM, respectively. In the HaCaT cells (moderate EGFR expression), the EC50 values for the anti-EGFR antibody, LAP-anti-EGFR antibody and MMP-2-activated LAP-anti-EGFR antibody were 2.4 nM, 18.1 nM and 2.1 nM, respectively. (**C**) The anti-EGFR antibody (), LAP-anti-EGFR antibody () and MMP-2-treated LAP-anti-EGFR antibody () were used to compete against a HA-tagged anti-EGFR antibody for binding to EGFR expressed on the cells. Bound HA-tagged anti-EGFR antibody was detected by an anti-HA antibody.

We next tested how binding kinetics of anti-EGFR antibodies were altered by LAP domains. Purified anti-EGFR antibodies and LAP-anti-EGFR antibodies were incubated with MMP-2 at 37 °C for 1 h and then tested by cell-based ELISA with the indicated dilution of the antibodies. Figure 4A shows that before MMP-2 treatment, the binding kinetic curve of LAP-anti-EGFR antibodies shifted to the right, whereas after incubation with MMP-2, the binding kinetic curve of LAP-anti-EGFR antibodies shifted to the left, to the levels similar to the unmodified anti-EGFR antibodies. The EC_50_ values of anti-EGFR antibody, LAP-anti-EGFR antibody and MMP-2-activated LAP-anti-EGFR antibody in EGFR-overexpressing MDA-MB-468 cells were 2.4 nM, 7.5 nM and 2.7 nM, respectively. In addition, we compared the binding activity of anti-EGFR and LAP-anti-EGFR antibodies using HaCaT keratinocyte cells, which express a moderate level of EGFR on cell surface (supplementary Figure 3). The binding kinetics of anti-EGFR antibodies were decreased by LAP domains in moderate EGFR-expressing HaCaT cells (EC_50_ 7.5-fold higher, Fig. 4B). The difference of EC_50_ in HaCaT cells was higher than the data obtained in the high EGFR-expressing MDA-MB-468 cells. This result is consistent with Carrido G. *et al*. who also showed that binding activity of an intermediate affinity anti-EGFR antibody (nimotuzumab) decreased when EGFR expression is decreased^26^. In conclusion, our results show that the LAP reduces the binding activity of the antibody in both high and moderate EGFR-expressing cells.

We further confirmed the specificities of anti-EGFR antibody and LAP-anti-EGFR antibody for binding to EGFR overexpressed on MDA-MB-468 cells. We used a HA-tagged anti-EGFR antibody as the readout for cell-based competition ELISA. Graded concentrations of anti-EGFR antibodies or LAP-anti-EGFR antibodies (with or without MMP-2 digestion) were mixed with a fixed amount of HA-tagged anti-EGFR antibody prior to addition to EGFR-overexpressing MDA-MB-468 cells, then we detected bound HA-tagged anti-EGFR antibody by a HA tag-specific antibody. As shown in Fig. 4C, increasing amounts of anti-EGFR antibodies indeed competed with the HA-tagged anti-EGFR antibody for binding sites on MDA-MB-468 cells as demonstrated by the proportionally decreased absorbance at 405 nm. However, untreated LAP-anti-EGFR antibodies did not significantly decrease the signal, indicating that LAP-anti-EGFR antibodies could not efficiently compete with HA-tagged anti-EGFR antibody. On the contrary, MMP-2-pretreated LAP-anti-EGFR antibodies proportionally decreased the absorbance, similar to the extent of the anti-EGFR antibodies. These results suggest that MMP-2-treated LAP-anti-EGFR antibodies can specific bind to the EGFR antigen expressed on the cells.

### The serum stability of LAP-anti-EGFR antibodies

**Figure 5:**
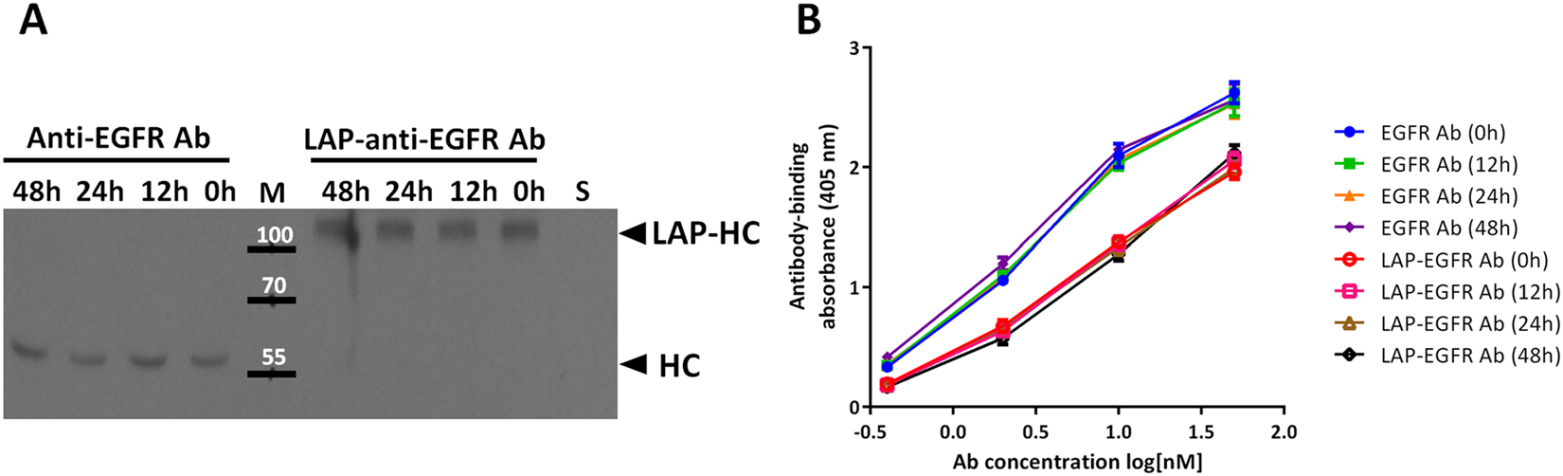
The serum stability of LAP-anti-EGFR antibodies. (**A**) Anti-EGFR antibodies and LAP-anti-EGFR antibodies were incubated with 20% fetal bovine serum at 4 °C for indicated time, then analyzed for protein stability by western blotting using anti-human Fcγ secondary antibodies. Incubations of anti-EGFR antibodies or LAP-anti-EGFR antibodies with serum (FBS) did not cause hydrolyses of the antibodies, as compared to the non-treated (0 h) group. S: serum alone. (**B**) Anti-EGFR antibodies and LAP-anti-EGFR antibodies were incubated with 20% fetal bovine serum at 4 °C for indicated time, then analyzed for binding activity by cell-based ELISA. Serum incubation of anti-EGFR antibodies and LAP-anti-EGFR antibodies did not alter binding activity. The original blot image for panel A is presented in Supplementary Figure S7.

We examined the serum stability of LAP-anti-EGFR antibodies *in vitro*. The anti-EGFR antibodies or LAP-anti-EGFR antibodies were incubated with 20% fetal bovine serum (FBS) at 4 °C for indicated time, then analyzed protein stability by western blotting, and binding activity by cell-based ELISA. Figure 5A shows that both of anti-EGFR antibodies and LAP-anti-EGFR antibodies are stable in serum. In addition, the binding activity were not affected after serum incubation throughout the indicated time (Fig. 5B). These results indicate that the LAP-anti-EGFR antibodies are proteolytically stable in serum.

### Applicability of LAP inhibitory domain in an anti-TNF-α antibody

**Figure 6:**
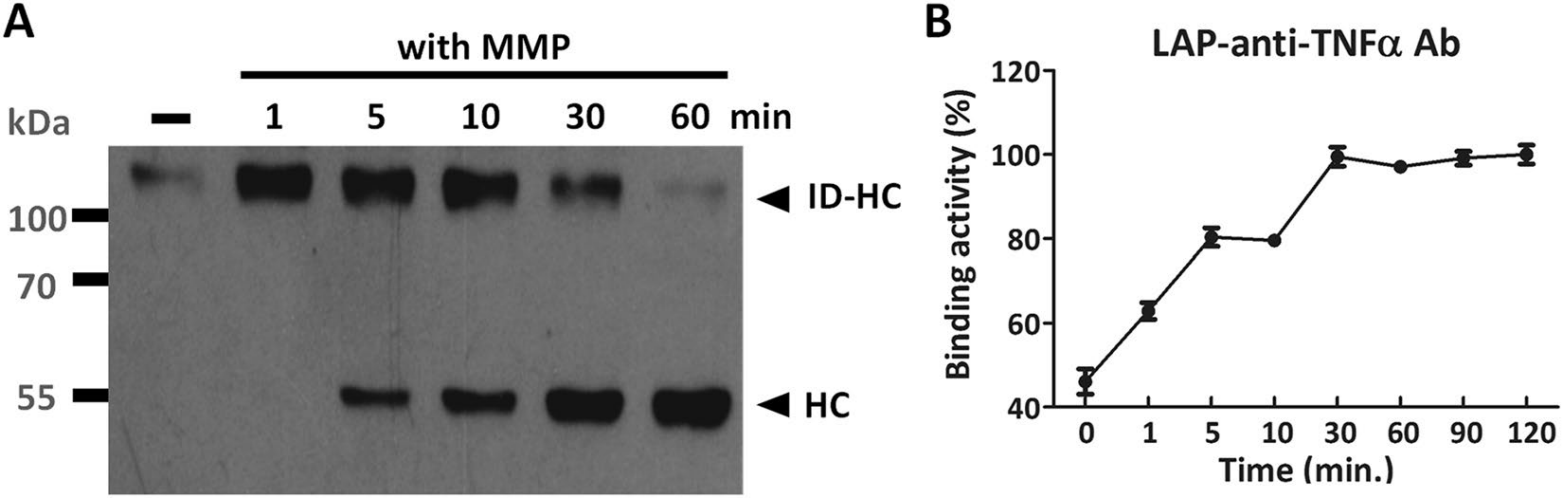
Applicability of LAP for masking an anti-TNF-α antibody. LAP-anti-TNF-α antibody was digested by MMP-2 for the indicated time and analyzed for removal of LAP by western blot (**A**) or revival of TNF-α binding activity by ELISA, as compared to the unmodified anti-TNF-α antibody (**B**). The LAP domain reduced antibody binding activity by 53.9%. Of note, after MMP-2 digestion of the LAP-anti-TNF-α antibody, the binding activity rose progressively to levels similar to that of the unmodified anti-TNF-α antibody. ID-HC: inhibitory domain-heavy chain. HC: heavy chain. with MMP: with MMP-2 incubation. The original blot image for panel A is presented in Supplementary Figure S8.

The strategy of masking antibody binding sites seemed to be most successful when using LAP. We therefore tested whether LAP can be used as a universal blocker for antibodies. To this end, we linked LAP to an anti-TNF-α antibody through the same MMP-2 substrate peptide. Similar to LAP-anti-EGFR antibody, the LAP domain can be effectively removed from the heavy chain of anti-TNF-α antibody by MMP-2 treatment in an incubation time-dependent fashion (Fig. 6A). The LAP reduced antibody binding to TNF-α by 53.9%, as compared to the unmodified anti-TNF-α antibody (Fig. 6B). Addition of MMP-2 to the recombinant antibody restored its binding activity to 100%, indicating MMP-2 can effectively remove the LAP inhibitory domain to revive the antibody function. These data indicate that linking LAP to a therapeutic antibody through a protease substrate peptide may be a useful strategy to render antibody function latent until it is activated by disease-associated proteases.

### Illustration of the masking efficiency of the inhibitory domains by computer molecular dynamic simulation

**Figure 7:**
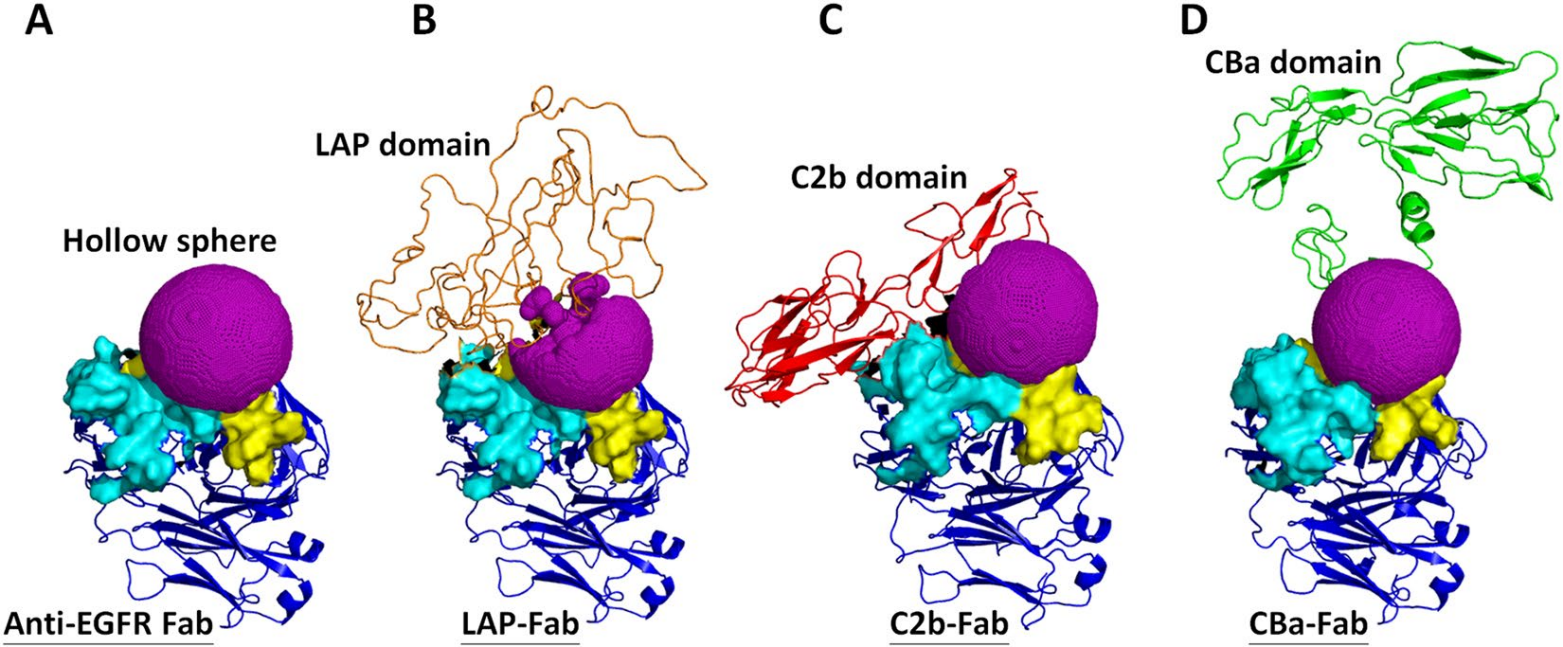
Structure simulation and masking efficiency analysis of pro-anti-EGFR antibodies. (**A**) Computer molecular dynamic simulation of anti-EGFR Fab structure with hollow sphere (purple). The CDRs of light chain (light blue) and heavy chain (yellow) are additionally marked to distinguish them from the Fab structure (blue). The protein structures of LAP-Fab (**B**), C2b-Fab (**C**) and CBa-Fab (**D**) are shown with hollow spheres (purple). A smaller sphere volume indicates a greater masking efficiency of the inhibitory domain. The sphere volumes were calculated to measure the masking efficiency of the LAP domain (orange), C2b (red) and CBa (green) over the binding sites of Fab.

We used computer molecular dynamic simulation to explore the possible mechanisms of the masking efficiency of inhibitory domains over the binding sites of antibodies (complementarity-determining regions, CDRs) (Fig. 3). We set the anti-EGFR Fab protein structure as main motif, then linked different inhibitory domains to the N-terminus of the heavy chain of an anti-EGFR Fab through a substrate peptide of MMP-2, then simulated the pro-anti-EGFR Fab by computer dynamic simulation. To explain the masking efficiency of inhibitory domains, we analyzed the spaces between CDRs and inhibitory domains. The program HOLLOW^27^ was used to calculate the channel spaces and interior spaces of protein structures by setting a sphere inside the structure then filled the sphere space with dummy atoms, and then to calculate the total numbers of atoms to obtain the sphere volume. We set a hollow sphere between the binding sites of the antibodies (CDRs) and the inhibitory domains (LAP/C2b/CBa), and then analyzed the masking efficiency of the inhibitory domains by calculating the volume of the hollow sphere (filled with oxygen atoms). A smaller sphere volume would indicate a greater masking efficiency of the inhibitory domain. Figure 7 shows the simulation of protein structures of anti-EGFR Fab (Fig. 7A), LAP-Fab (Fig. 7B), C2b-Fab (Fig. 7C) and CBa-Fab (Fig. 7D) with hollow sphere. The sphere volume of the LAP-Fab was smallest (filled with 3980 oxygen atoms), followed by the C2b-Fab (5382 oxygen atoms) and finally the CBa-Fab (6322 oxygen atoms). According to these calculations (Table 1), the masking efficiency of the LAP domain, C2b domain and CBa domain were 33.7%, 10.3% and −5.4%, respectively. These results suggested that the LAP domain covered the CDR most extensively among the three different inhibitory domains, which is in line with our data showing reduced binding activities of pro-anti-EGFR antibodies imposed by the inhibitory domains (Fig. 3). Thus the computer simulation can be used to explain the masking efficiency of inhibitory domains over the binding sites of antibodies.

**Table 1:** Calculation of the masking efficiency of inhibitory domains.

Antibody	Numbers of oxygen atom of sphere (N)	The volume of sphere (S = N × V) (m^3^)	% S	% M
EGFR Fab	5999	8.82087 × 10^−26^	100%	0
LAP-EGFR Fab	3980	5.85215 × 10^−26^	66.3%	33.7%
C2b-EGFR Fab	5382	7.91364 × 10^−26^	89.7%	10.3%
CBa-EGFR Fab	6322	9.29581 × 10^−26^	105.4%	−5.4%

NOTE: The volume of sphere (S) is the number of oxygen atoms (N) multiplied by the density of oxygen atoms (V = 1.47039 × 10^−29^(m^3^)). % S, the percentage of sphere volume = 100 × (S_pro-Fab_/S_Fab_). % M, the percentage of masking efficiency = % S_Fab_ − % S_pro-Fab_.

## Discussion

Systemic injection of antibodies may cause considerable adverse effects. We used an inhibitory domain to mask the binding sites of antibodies to develop MMP-2-activated pro-antibodies as a strategy to reduce systemic targeting and adverse effects of the antibodies. This strategy takes advantage of the fact that many proteases such as MMP-2 are selectively upregulated in disease sites^28^. Using this strategy, inactive pro-antibodies may be selectively converted into active antibodies at the disease sites by MMP-2, while leaving the target molecules in normal tissues untouched. We demonstrated that N-terminally linked LAP can indeed mask the binding sites of the recombinant LAP-anti-EGFR antibody and LAP-anti-TNF-α antibody. Cleavage of LAP domain from the recombinant antibodies revived the binding activities comparable to that of their unmodified forms. Our finding that LAP can mask the binding sites of two antibodies suggests that LAP may be applicable to other antibodies to generate disease site-selective pro-antibodies. Thus LAP-masked pro-antibody is a promising strategy for the development of personalized protease targeted therapies.

With regard to the relationship between the reduction of binding activity and the reduction of the on-target toxicity of antibody, Desnoyers and coworkers showed that a peptide-masked EGFR probody displaying a higher EC_50_ (than the parental antibody; 48-fold difference) could improve the safety of therapeutic antibodies in nonhuman primates^29^; Yang and coworkers also demonstrated that a probody with 20-fold weaker binding activity (than that of parental antibody) displayed a lower targeting in adjacent normal tissues^30^; Donaldson and coworkers also proved a peptide-masked scFv with lower binding affinity (eight-fold lower than unmasked scFv) displayed a lower targeting to keratinocyte-derived HaCaT cells^31^. Finally, Crombet and coworkers pointed out that lowering the affinity between a monoclonal anti-EGFR antibody (nimotuzumab) and EGFR reduced on-target toxicity to normal cells. Their mathematical models predict that the binding affinity (K_D_) for anti-EGFR antibodies should be in the range of 10^−8^ M–10^−9^ M to maximize tumor cell targeting while minimizing normal cell toxicity^32^. It is therefore concluded that lowering binding activity in terms of EC_50_ or K_D_ may reduce on-target toxicity to normal tissues. In our study, the EC_50_ of LAP-anti-EGFR antibody is 3.1-fold higher than the parental anti-EGFR antibody (Fig. 4A), thus we expect LAP may reduce the on-target toxicity of antibodies in normal tissue.

Several strategies have been attempted to design pro-antibodies; however, technical challenges remain. For example phage display technology was used to select peptide capable of masking the binding sites of anti-EGFR antibodies^29, 33, 34^. These phage display-selected peptides are likely non-endogenous and may provoke neutralizing antibodies once injected to humans, thus limiting their long term use in humans. In addition to phage display technology, other investigators used epitope sequences recognized by the antibody (such as anti-EGFR antibody) as masking peptides^31^. However, the epitope peptides bind to the antibody with high affinity (i.e., the nature of antibody-antigen binding), such that it may remain bound after proteases have cleaved the linker between the antibody and the peptides. Furthermore, the affinity-based approach requires search and selection of a unique phage display-selected peptide/epitope peptide for each antibody, making generalized application of a selected peptide to other antibodies difficult.

To address these technical challenges, we used the concept of steric hindrance to select inhibitory domains from endogenous proteins. Thus LAP-masked pro-antibodies are expected to have low immunogenicity and are safer for use in humans. On the other hand, the masking peptide LAP can dissociate from the antibodies once the intervening linker is cleaved by the protease. This notion is supported by our data that binding activity of our pro-antibodies can be revived upon treatment with MMP-2. Considering that all antibodies are composed of light chains and heavy chains and that different antibodies share a very similar three dimensional structure, grafting the LAP domain to other antibodies may impose similar steric hindrance to their binding sites. Thus the LAP domain may represent an efficient and rapid way to generate protease-activated pro-antibodies.

Proteases play an important role in the progression of many diseases^35^. Proteases cleave many different targets (extracellular matrix, cytokines, growth factors and cytokine/growth factor receptors that modulate pathophysiology in diseases^28, 36, 37^. High expression of proteases is a characteristic of many diseases. Given their differential expression patterns at disease sites and in normal tissues^38^, proteases are attractive molecular targets for the design of new therapies or diagnoses that act preferentially at disease sites while leaving normal tissues untouched. Indeed, several reports have engineered molecular probes^39, 40^ or protease-activated prodrugs suited for this aim^29, 41^. For example Desnoyers *et*. *al*. have developed an EGFR-specific probody that is activated by the urokinase-type plasminogen activator (uPA) and membrane-type serine protease 1 (MT-SP1)^29^. We choose to target MMP-2 in our proof-of-concept test of protease-activated pro-antibodies. This concept, however, is not limited to MMP-2 but may be further expanded for other protease-related diseases by replacing the substrate linkers between the inhibitory domains and targeting antibodies. For example, cysteine protease cathepsins^36^ and legumain^37^ are well-known proteases that are overexpressed in some tumors or arthritis sites. Specific linker substrates can be tailor-made to suit patients’ protease profiling at the disease sites to achieve personalized medicine.

Our computer molecular dynamic simulation shows that the LAP inhibitory domain masked the binding sites of antibodies most extensively. This result is consistent with the inhibition of binding activity before MMP-2 activation (Fig. 3). However, many of the selected inhibitory domains failed in our test possibly due to distorted conformations when linked to the antibodies. We consider that the linker between inhibitory domains and antibodies was too flexible to lose direction, so it may cause ineffective masking of the antibodies. Further study to modify the linker sequences between the inhibitory domain and antibody binding sites via simulation of molecular dynamics may reveal the optimized linker for each antibody. Though computer-simulation may not reveal real protein structures, it can provide insights to rational design and improve developmental efficiency of protease-activated pro-antibodies. In the future, crystallography may be required to solve the definitive structures of pro-antibodies to fully explain the mechanism on how LAP masks the binding activities of the antibodies.

In summary, LAP domain-masked, MMP-2-activated, pro-antibodies possess the following advantages: (1) pro-antibodies can increase disease site selectivity, thus reducing systemic adverse effects, by targeted activation of the pro-antibodies by proteases over-expressed at disease sites; (2) pro-antibodies have low immunogenicity due to the endogenous origin of the inhibitory domain; (3) steric hindrance of the inhibitory domain can be easily removed from the pro-antibodies once the linker is cleaved by the protease; (4) the LAP inhibitory domain can be adopted for other antibodies; and (5) the linker substrate can be changed to suit other disease-associated proteases. According to these advantages, the LAP domain may be useful for the development of novel pro-antibodies. This strategy may improve the safety of the antibodies and likely increase patient compliance to achieve a better therapeutic index.

## Materials and Methods

### Cell culture

Human embryonic kidney 293 cells stably expressing large-T antigen (293 T) and MDA-MB-468 human breast cancer cells were cultured in Dulbecco’s modified Eagle’s medium (DMEM, Sigma-Aldrich, St. Louis, MO, USA) supplemented with 10% cosmic calf serum (CCS, HyClone, Logan, UT, USA), 100 units/mL penicillin, and 100 μg/mL streptomycin (Gibco Laboratories, Grand Island, NY, USA) at 37 °C in a humidified atmosphere of 5% CO_2_. The Expi293 cells were grown in Expi293 expression medium (Gibco Laboratories, Grand Island, NY, USA) and cultured in shake flasks under standard procedures at 120 rpm and 37 °C in an incubator with 8% CO_2_.

### Plasmid construction of pro-antibody

A schematic representation of the pro-antibody expression constructs is shown in Fig. 1B. To construct the expression vectors encoding the full-length anti-EGFR antibody and anti-TNF-α antibody, a variable fragment of human anti-EGFR antibody was cloned based on the h528Fv DNA sequence^42^ and a variable fragment of human anti-TNF-α antibody was cloned based on the Remicade sequence (from US Patent 7,070,775) by assembly PCR. The VH-CH1 domain of the antibody, assembled with the human IgG1 Fc domain via assembly PCR, was cloned into the pLHCX mammalian expression vector to acquire the human Igκ leader sequence (LS) at the N terminus of the antibody heavy chain expression sequence. The VL-Cκ domain of antibody with a leader sequence was inserted at the N terminus and a furin-2A (F2A)-based bicistronic sequence^43^ was cloned into the pLHCX-LS-anti-EGFR/TNF-α heavy chain expression vector to form an anti-EGFR/TNF-α antibody expression cassette. The coding sequences of complement factor 2, complement factor B and latency-associated peptide genes were purchased from TransOMIC Technologies (Huntsville, AL, USA). The LAP and C2b of C2 and CBa of CB fragments-containing MMP-2 substrate peptide^44^ were cloned and amplified by PCR, and then inserted into the antibody expression cassette, between the F2A and the heavy chain of the antibody. The MMP-2 substrate peptide (GPLGVR)-linked anti-EGFR antibodies heavy chain were cloned and amplified by PCR, and then replaced the heavy chain to GPLGVR-heavy chain in the antibody expression cassette.

### Transient expression and purification of pro-antibody

293 T cells (1.5 × 10^6^ cells/well) were seeded in 6-well plate contained in DMEM supplemented with 10% cosmic calf serum and placed in an incubator at 37 °C overnight. The cells were transfected with 2.5 μg pLHCX-pro-anti-EGFR or pLHCX-anti-EGFR plasmids by TransIT-LT1 transfection reagent (Mirus Bio Corporation, Madison, WI, USA) and cultured in DMEM serum-free medium for 48 hours. The cells and media were collected for subsequent experiments. Alternatively, 6 × 10^7^ Expi293 cells in suspension were transfected with 30 μg pLHCX-LAP-anti-TNF-α or pLHCX-anti-TNF-α plasmids by the Expifectamine reagent (Life Technologies, Carlsbad, CA, USA). Cells were cultured in Expi293 media with enhancers as specified by the manufacturer. The media were harvested for subsequent experiments 5 days post-transfection. The anti-EGFR antibodies and LAP-anti-EGFR antibodies were purified by protein A sepharose 4 Fast Flow chromatography (GE Healthcare, Little Chalfont, UK). The purity of purified anti-EGFR antibodies and LAP-anti-EGFR antibodies were analyzed by SDS-PAGE in reducing and nonreducing conditions (supplementary Figure 4).

### MMP-2 activation

The pro-antibody or antibody samples were incubated with 5 μg/ml MMP-2 (type IV collagenase, Sigma-Aldrich, St. Louis, MO, USA) or buffer only (DMEM) for different incubation times (1, 5, 10, 30, 60, 90 and 120 minutes). In our experiments, we opted to use a higher concentration of MMP-2 (1 μg/200 μL, 2700 μU/μg solid enzymes; μU, Furylacryloyl-Leu-Gly-Pro-Ala (FALGPA) peptide hydrolysis activity in microunits) and a shorter digestion time (1 h) as compared to prolonged digestion by lower concentrations of MMP-2 in the colorectal cancer tumor tissues (4762.5 μU/mg protein)^45^. After MMP-2 digestion, samples were mixed with 6× sodium dodecyl sulfate (SDS) reducing loading dye for gel electrophoresis and western blotting analyses, or mixed with 10% cosmic calf serum for ELISA experiments.

### Analyses of MMP-2 digestion of pro-Abs by western blotting

The pro-antibodies and antibodies with or without MMP-2 treatment were mixed with 6× SDS reducing loading dye and boiled for 10 min. Samples were separated by 10% SDS-PAGE (sodium dodecyl sulfate-polyacrylamide gel electrophoresis) and then transferred to nitrocellulose (NC) membranes (Millipore, Billerica, MA, USA). After blocking with PBS containing 5% milk at 4 °C overnight, the membranes were incubated with HRP-conjugated goat anti-human IgG Fcγ antibodies (Jackson ImmunoResearch Laboratories, West Grove, PA, USA) at room temperature for 1 hour. After washing in PBST (PBS containing 0.05% Tween 20) three times and in PBS once, the blots were developed by ECL reagent (Millipore, Billerica, MA, USA) and chemiluminescence was detected on autoradiography films.

### Analyses the protein aggregation of anti-EGFR antibodies and LAP-anti-EGFR antibodies by size-exclusion high-performance liquid chromatography

Anti-EGFR antibodies and LAP-anti-EGFR antibodies (100 μg/sample) were injected into an Aligent Bio SEC-5 column (300 × 7.8 mm, 300 Å) and separated at 1 ml/min in 50 mM sodium phosphate buffer, pH7.0. The protein peaks were detected at 280 nm.

### Immunoprecipitation of the MMP-2-treated LAP-anti-EGFR antibodies

Purified GPLGVR-anti-EGFR antibodies (MMP-2 substrate peptide-linked anti-EGFR Abs) and LAP-anti-EGFR antibodies were incubated with 5 μg/ml MMP-2 or buffer only (DMEM) at 37 °C for 1 h. The MMP-2 reaction were stopped with 15 mM EDTA and then immunoprecipitated by protein A sepharose beads with rotation at room temperature for 30 minutes. The beads were pelleted from the mixture by centrifugation and washed six times by PBS. The beads were mixed with 6× sodium dodecyl sulfate (SDS) sample loading buffer for SDS-PAGE and then the gel stained with Coomassie brilliant blue staining solution.

### Analyses of the binding activities of the MMP-2-treated pro-anti-EGFR Abs by cell-based ELISA

EGFR-overexpressing MDA-MB-468 cells and moderate EGFR-expressing HaCaT cells (10^5^ cells/well) were grown overnight in 96-well microtiter plates pre-coated with 40 μg/mL of poly-L-lysine (50 μL/well). After removing the original culture medium from the 96-well plates, the cells were washed with DMEM (200 μL/well) three times, the indicated dilution of anti-EGFR antibodies or MMP-2 pretreated or untreated pro-anti-EGFR antibodies were added to the cells (50 μL/well) at room temperature for 2 hours. After the cells were washed with DMEM (200 μL/well) three times to remove unbound antibodies, 1 μg/ml of HRP-conjugated goat anti-human IgG Fcγ antibodies (Jackson ImmunoResearch Laboratories) were added to the cells (50 μL/well) at room temperature for 1 hour. Finally, after washing with DMEM three times and PBS one time, ABTS substrates were added for 30 min before optical absorbance values at 405 nm were measured using an ELISA reader (Molecular Devices, Menlo Park, CA, USA). The EC_50_ for antibodies were analyzed by nonlinear regression curve fitting using the computer program GraphPad Prism 6.0 (GraphPad Software, San Diego, CA, USA). Data are presented as the mean of three independent experiments.

### Examination of the expression level of EGFR on the cell surface by flow cytometry

EGFR expression were measured by staining the cells with 1 μg/mL Erbitux in PBS containing 0.05% bovine serum albumin (BSA) for 50 minutes at 4 °C, followed by 1 μg/mL FITC-conjugated goat anti-human IgG Fc (Jackson ImmunoResearch Laboratories, West Grove, PA) for 50 minutes at 4 °C. After extensive wash in ice-cold PBS containing 0.05% BSA, the surface immunofluorescence of viable cells was measured with a FACScan flow cytometer (Beckman Coulter FC500 Cytometer) and fluorescence intensities were analyzed with CXP software (Beckman Coulter).

### Examination of the specificity of anti-EGFR antibodies and LAP-anti-EGFR antibodies by competition cell-based ELISA

EGFR overexpressing MDA-MB-468 cells (10^5^ cells/well) were seeded in 96-well microtiter plates as above. Serially diluted anti-EGFR antibodies, untreated or MMP-2 pretreated LAP-anti-EGFR antibodies were prepared and mixed 1:1 (v/v) with 50 nM of HA-tagged anti-EGFR antibody, and then the mixture were added to the cells at room temperature for 1 h. After proper washing in PBS, cells were sequentially incubated with HRP-conjugated anti-HA antibodies and ABTS at room temperature. The optical absorbance were measured at 405 nm using an ELISA reader.

### Examination of the serum stability of LAP-anti-EGFR antibodies *in vitro*

The anti-EGFR antibodies or LAP-anti-EGFR antibodies were incubated with 20% fetal bovine serum (FBS, Biological Industries) at 4 °C for 0 h, 12 h, 24 h or 48 h, then a proportion of the incubations were mixed with 6× sodium dodecyl sulfate (SDS) reducing loading dye for gel electrophoresis and western blotting analyses. The remaining incubations were serially diluted and tested for binding to EGFR by cell-based ELISA as described above.

### Analysis of the binding activities of the MMP-2-treated LAP-anti-TNF-α antibodies by ELISA

ELISA microplates (96-well, Maxisorp, Nunc, Roskilde, Denmark) were coated with 1 μg/mL of TNF-α (R&D Systems, Minneapolis, MN, USA) in 0.1 M NaHCO_3_ (pH8.3) coating solution (50 μL/well) for 2 hours at 37 °C and then blocked with PBS containing 5% skim milk (200 μL/well) for 2 hours at 37 °C. The anti-TNF-α antibody or MMP-2 pretreated or untreated pro-anti-TNF-α antibodies were added to the wells (50 μL/well) at room temperature for 2 hours. After washing with PBST (PBS containing 0.05% Tween 20) three times and PBS once to remove unbound antibodies, 1 μg/ml of HRP-conjugated goat anti-human IgG Fcγ antibodies were added to the wells (50 μL/well) at room temperature for 1 hour. After extensive washing as above, ABTS substrate was added for 30 min before optical absorbance at 405 nm was measured using an ELISA reader.

### Computer molecular dynamic simulation of pro-antibodies

The anti-EGFR Fab were built by Homology modeling^46^, using the antibody against foot-and-mouth disease virus (Protein Data Bank (PDB) ID entry 1QGC) as a template. The LAP, C2b and CBa domain were extracted from pro-TGF-β1 (PDB ID 3RJR), complement factor 2 (PDB ID 3ERB) and complement factor B (PDB ID 2OK5) by Swiss-PDB viewer^47, 48^, respectively. Then, the MMP-2 substrate linker (GGGGS-GPLGVR) were inserted into the C terminus of inhibitory domains by edit protein sequence using Discovery Studio 3.0 software. After insertion of the MMP-2 substrate linker into the C terminus of inhibitory domains, the inhibitory domain-linker was ligated to the N terminus of the heavy chain of anti-EGFR Fab by Swiss-PDB viewer. The structure of the pro-anti-EGFR Fab was built by homology modeling and dynamically simulated with the program Nanoscale Molecular Dynamics (NAMD)^49^, then illustrated by Pymol. The anti-EGFR Fab complementarity-determining regions (CDRs) of the light chain and heavy chain were defined by using the Kabat amino acid numbering scheme^50^.

### Calculation of masking efficiency of the inhibitory domain of the pro-antibody

The masking efficiency of the inhibitory domains over the binding sites of antibodies were calculated with the program HOLLOW^27^. In this program, a hollow sphere above the binding sites of antibodies (CDRs) was set as a tool to calculate the space between CDRs and the inhibitory domain. The highest amino acid (57S, located at the heavy chain CDR2) of anti-EGFR Fab was selected as the center of the hollow sphere, and then a sphere was drawn with a 12 Å radius, which is the cutoff distance of non-bonded interactions (e.g. electrostatic interactions or wan der Waals forces); distance greater than this range the non-bonded interactions are assumed to zero^51^. A smaller sphere volume indicates a smaller space between CDRs and the inhibitory domain, showing the masking efficiency of the inhibitory domain was effective. To calculate the sphere volume of pro-anti-EGFR Fab (LAP-Fab/C2b-Fab/CBa-Fab), the space of the sphere was filled with oxygen atoms. The number of oxygen atoms (N) was multiplied by the density of the atoms (V) to derive sphere volume (S) by the following formula:

$${\rm{S}}={\rm{N}}\times {\rm{V}}$$

The density of oxygen atoms was calculated by volume of sphere formula (V = πr^3^ × 4/3). The radius of oxygen atom is 1.52 Å. The sphere volume of anti-EGFR Fab (S_Fab_) was set as 100%. Results were expressed as a percentage of sphere volume of pro-Fab (LAP-Fab/C2b-Fab/CBa-Fab) (S_pro-Fab_) compared to the S_Fab_ by the following formula: The percentage of sphere volume (% S) = 100 × (S_pro-Fab_/S_Fab_). The masking efficiency was calculated by the following formula: The percentage of masking efficiency (% M) = % S_Fab_ − % S_pro-Fab_.

## Electronic supplementary material

supplementary information
