# Supplementary material for: Selective antibody activation through protease-activated pro-antibodies that mask binding sites with inhibitory domains
Source: Sci Rep. 2017 Sep 14;7:11587. doi: 10.1038/s41598-017-11886-7 (PMC5599682; doi:10.1038/s41598-017-11886-7)
Supplement: Supplementary file 1 — supplementary information [file 41598_2017_11886_MOESM1_ESM.pdf]

Supplementary information

Selective antibody activation through protease-activated pro-antibodies that mask binding sites with inhibitory domains

I-Ju Chen[1,#], Chih-Hung Chuang[2,3,#], Yuan-Chin Hsieh[1], Yun-Chi Lu[1], Wen-Wei Lin[4], Chien-Chiao Huang[3,5,6], Ta-Chun Cheng[3], Yi-An Cheng[1], Kai-Wen Cheng[4], Yeng-Tseng Wang[7], Fang-Ming Chen[8], Tian-Lu Cheng[1,3,4,6,9,*] and Shey-Cherng Tzou[10,**]

[1] Graduate Institute of Medicine, Kaohsiung Medical University, Kaohsiung, Taiwan

[2] Department of Medical Laboratory Science and Biotechnology, College of Health Sciences, Kaohsiung Medical University, Kaohsiung, Taiwan

[3] Center for Biomarkers and Biotech Drugs, Kaohsiung Medical University, Kaohsiung, Taiwan

[4] Institute of Biomedical Sciences, National Sun Yat-Sen University, Kaohsiung, Taiwan

[5] Graduate Institute of Clinical Medicine, Kaohsiung Medical University, Kaohsiung, Taiwan

[6] Department of Biomedical Science and Environmental Biology, Kaohsiung Medical University, Kaohsiung, Taiwan

[7] Department of Biochemistry, Kaohsiung Medical University, Kaohsiung, Taiwan

[8] Department of Surgery, Faculty of Medicine, College of Medicine, Kaohsiung Medical University, Kaohsiung, Taiwan

[9] Department of Medical Research, Kaohsiung Medical University Hospital, Kaohsiung, Taiwan

[10] Institute of Molecular Medicine and Bioengineering, Department of Biological Science and Technology, National Chiao Tung University, Hsin-Chu, Taiwan

[#] These authors contributed equally to this work.

[*] Corresponding author: Department of Biomedical Science and Environmental Biology, Kaohsiung Medical University, 100 Shih-Chuan 1st Road, Kaohsiung, 80708 Taiwan. Tel.: +886 7 3121101 2697. Fax: +886 7 3227508. E-mail: tlcheng@kmu.edu.tw

[**] Corresponding author: Institute of Molecular Medicine and Bioengineering, Department of Biological Science and Technology, National Chiao Tung University, 75 Bo-Ai Street, Hsin-Chu, 30068 Taiwan. Tel: +886 3 5712121 Ext 56904, Fax: +886 3 5729288. E-mail: sctzou@gmail.com

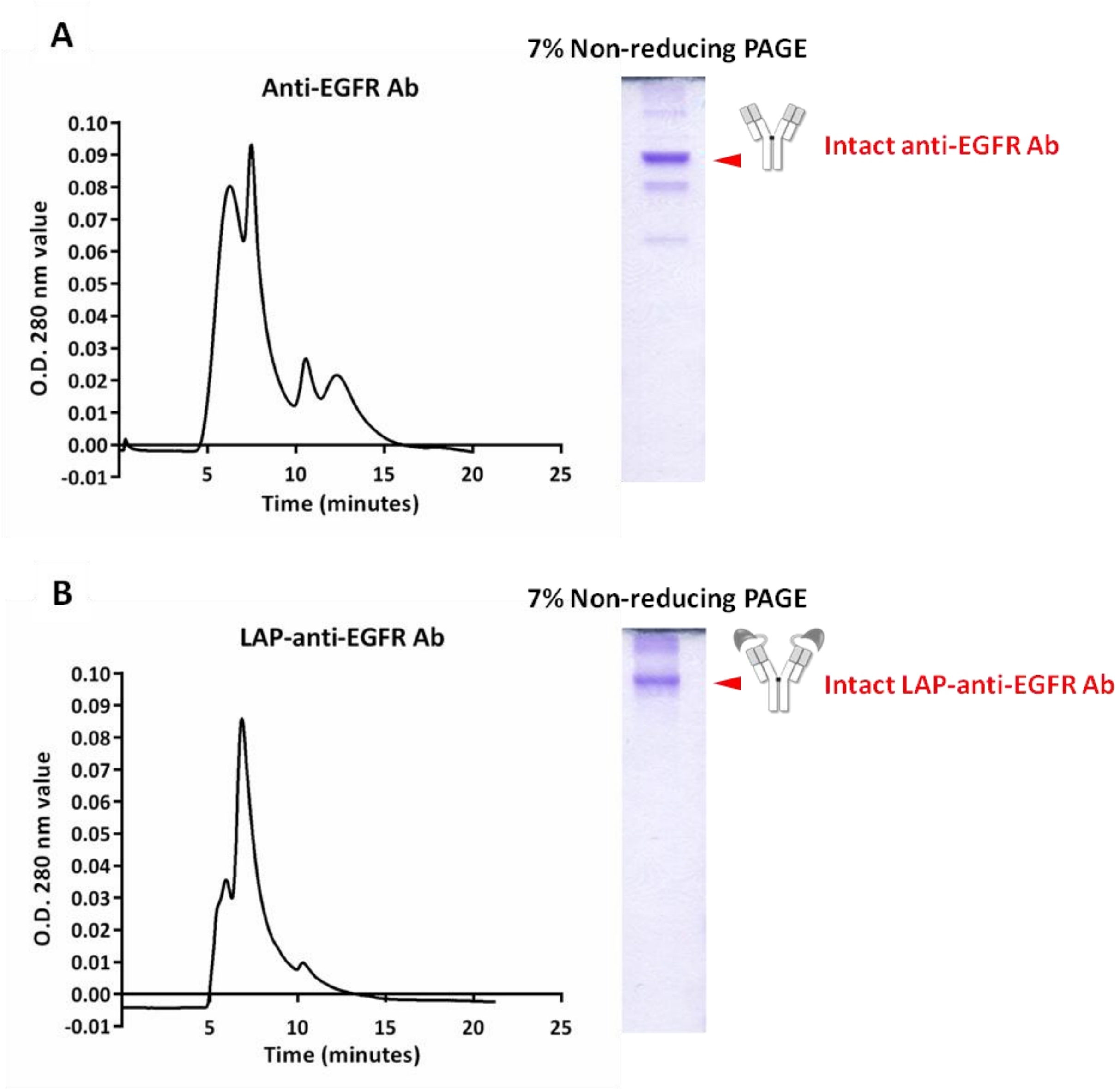

Figure S1: Size-exclusion high-performance liquid chromatography of anti-EGFR antibodies and LAP-anti-EGFR antibodies.

The patterns of size-exclusion chromatography analyses (left panel) are similar between (A) anti-EGFR antibodies and (B) LAP-anti-EGFR antibodies. A major peak was detected in HPLC that matches the major band in Coomassie blue staining (right panel), indicating the monomeric conformation. A preceding minor peak in the HPLC analyses may indicate aggregate of the anti-EGFR antibodies and LAP-anti-EGFR antibodies. The original gels image for panel A and B are presented in Supplementary Figure S10.

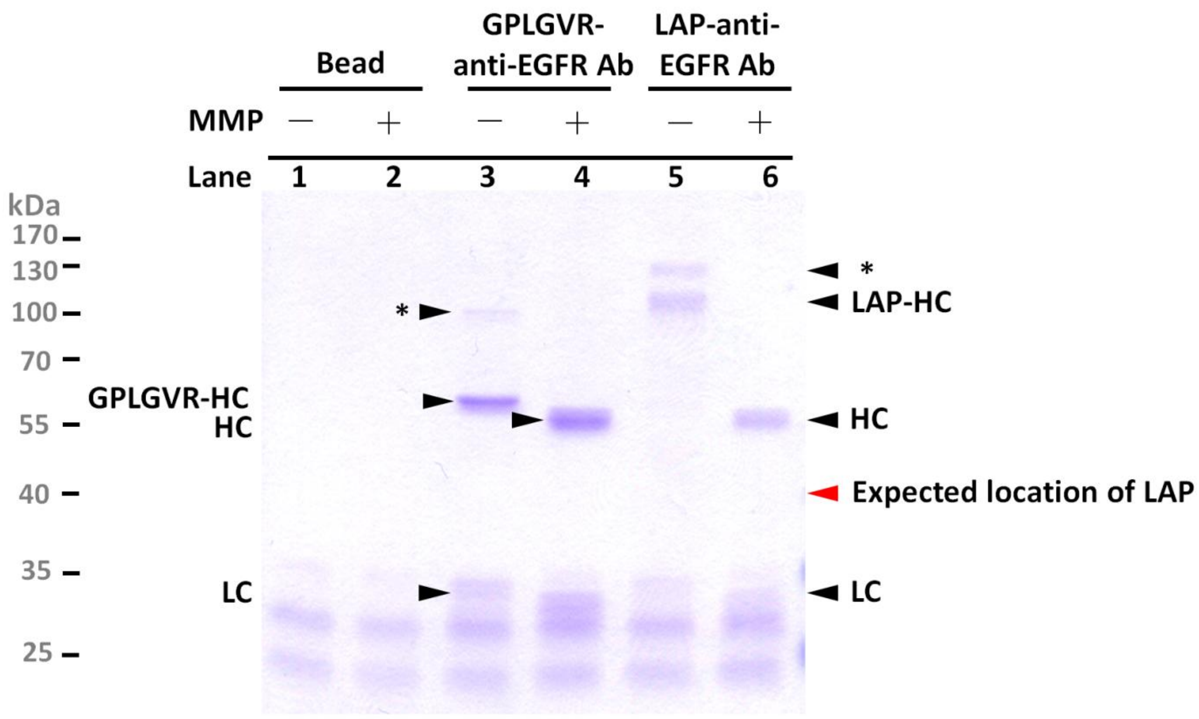

Figure S2: LAP domains are not bound to anti-EGFR antibody.

Purified GPLGVR-anti-EGFR (substrate peptide-linked anti-EGFR) antibodies and LAP-anti-EGFR antibodies were incubated with MMP-2 or buffer only (DMEM) at 37℃ for 1h, then precipitated by protein A sepharose beads. The samples were resolved by reducing SDS-PAGE and then stained in gel with Coomassie brilliant blue. Locations of each recombinant protein are indicated. Asterisks (*) indicate the light chain (LC) still fused with heavy chain (HC) or LAP-heavy chain (LAP-HC) due to incomplete cleavage on furin-2A sequence in the cells. The location of LAP domain in the gel is indicated by a red arrowhead. No ~40 kDa (the molecular weight of LAP) protein band was noted in MMP-2 treated LAP-anti-EGFR antibodies in the gel (lane 6), indicating LAP domains were not associated with the anti-EGFR after MMP-2 digestion. Bead: protein A sepharose beads alone. Light chains of antibodies can be cleaved at the very N-terminus by MMP-2 (unpublished data), rendering a slight reduction on the molecular weight. However, cleavage at the very N-terminus does not affect antibody function as in the case of the EGFR antibodies. The original gel image is presented in Supplementary Figure S9.

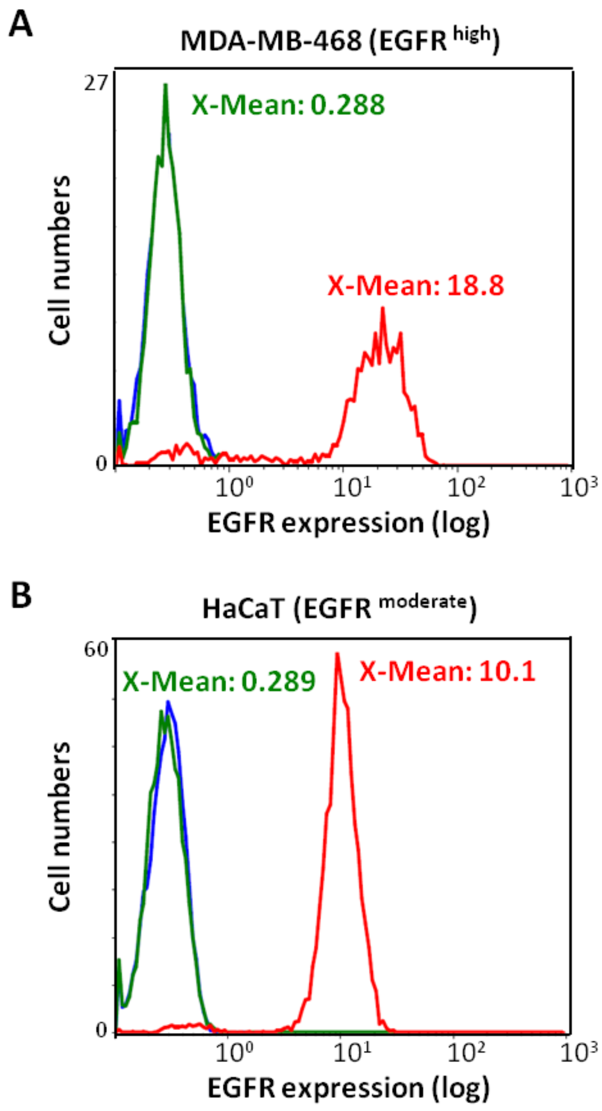

Figure S3: The expression level of EGFR in MDA-MB-468 and HaCaT cells.

(A) MDA-MB-468 breast cancer cells and (B) HaCaT keratinocyte cells were analyzed by staining the cells with Erbitux (anti-EGFR antibodies), followed by FITC-conjugated goat anti-human IgG Fc. The cell surface expressions of EGFR were divided into high and moderate expression based on the X-Mean (mean fluorescence intensity). Cell alone control without any staining (blue line), cells stained by FITC-conjugated goat anti-human IgG Fc alone (green line), cells stained by Erbitux + FITC-conjugated goat anti-human IgG Fc (red line).

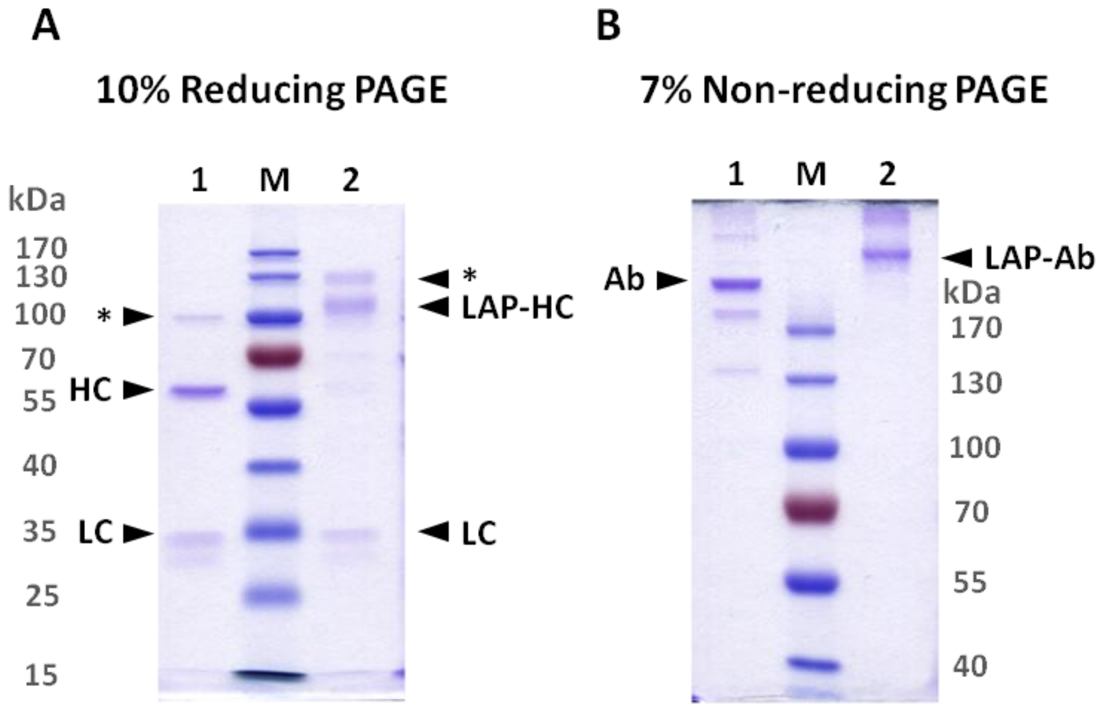

Figure S4: SDS-PAGE analysis of purified anti-EGFR antibodies and LAP-anti-EGFR antibodies.

SDS-PAGE analysis of purified anti-EGFR antibodies and LAP-anti-EGFR antibodies under reducing (A) and non-reducing (B) conditions. Lane 1: anti-EGFR antibodies; Lane 2: LAP-anti-EGFR antibodies; M: Protein Marker. Under reducing conditions, asterisks (*) indicate the light chain (LC) was still fused with heavy chain (HC) or LAP-heavy chain (LAP-HC) due to incomplete cleavage on furin-2A sequence in the cells. Under non-reducing conditions, the full anti-EGFR antibodies and LAP-anti-EGFR antibodies are indicated by arrowheads. The original gels image for panel A and B are presented in Supplementary Figure S10.

Original gel/blot images

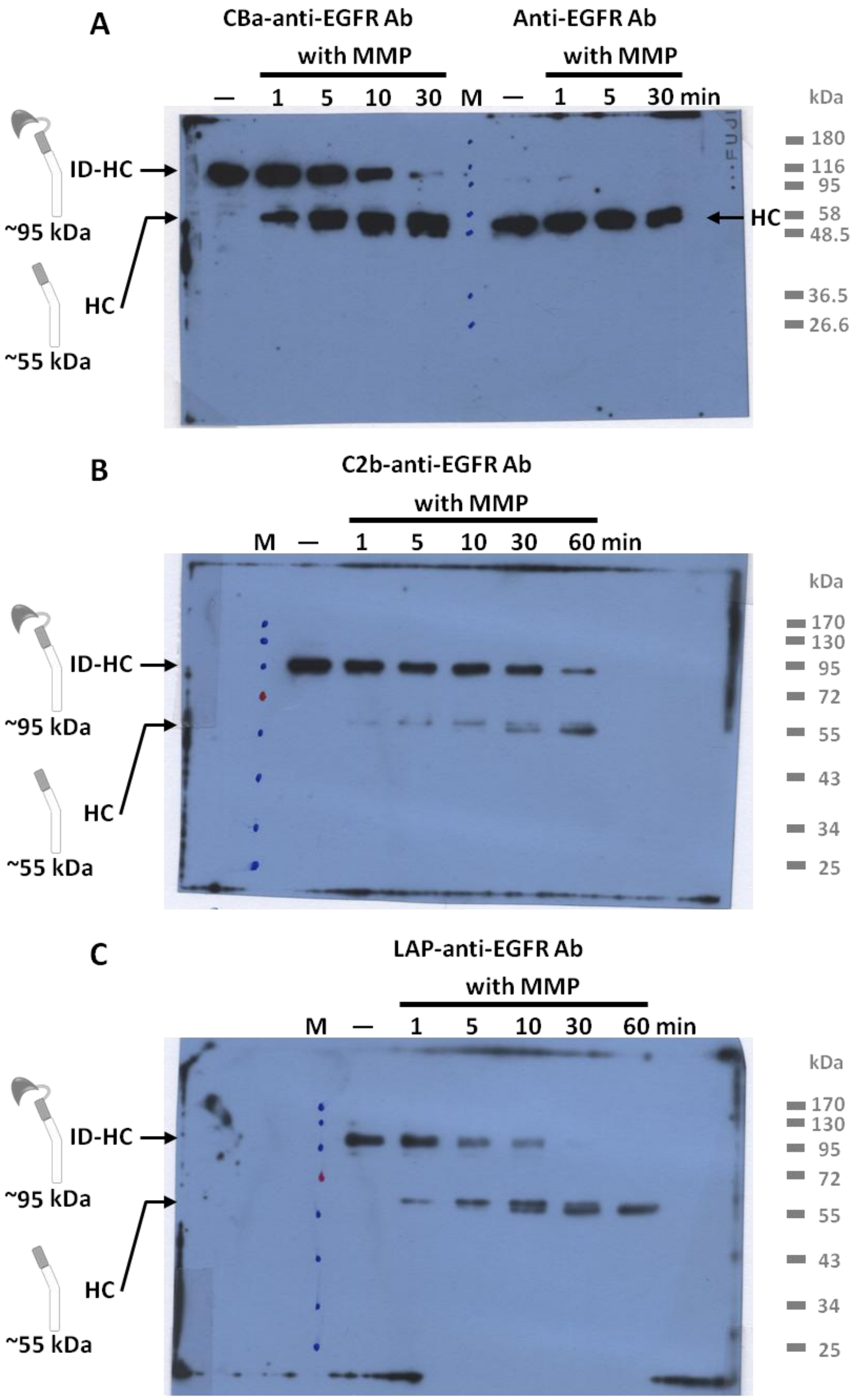

Figure S5: The original blots for cropped blots presented in Figure 2.

(A) The original blot for panel A (right) and B (left) presented in Figure 2 in the main text. (B) The original blot for panel C presented in Figure 2 in the main text. (C) The original blot for panel D presented in Figure 2 in the main text. ID-HC: inhibitory domain-heavy chain. HC: heavy chain. with MMP: with MMP-2 incubation. M: Protein marker.

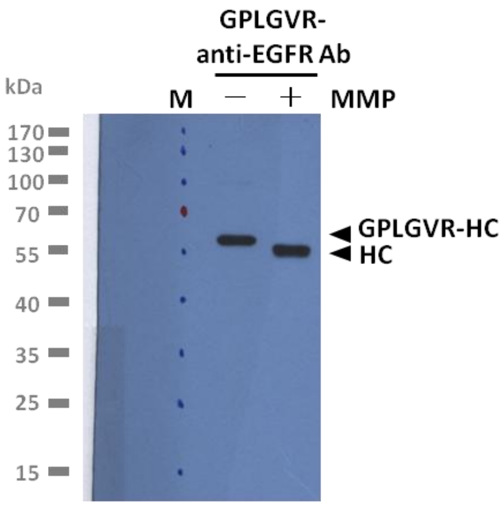

Figure S6: The original blot for blot presented in Figure 3E.

GPLGVR-HC: MMP-2 substrate peptide-linked heavy chain. HC: heavy chain. M: Protein marker.

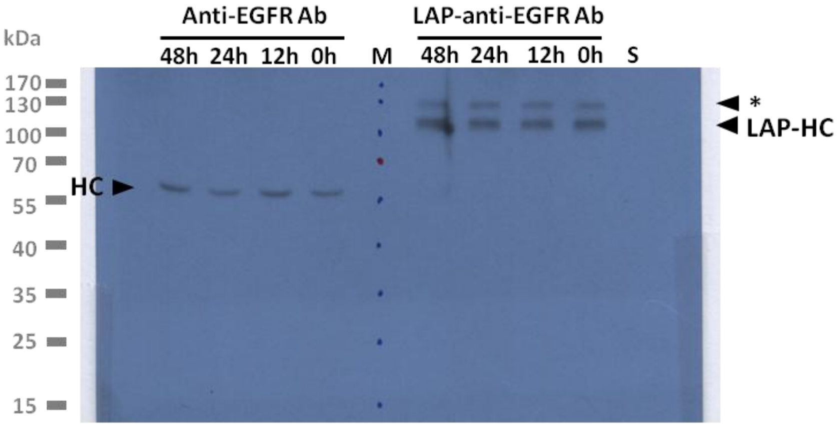

Figure S7: The original blot for cropped blot presented in Figure 5A.

Asterisks (*) indicate the light chain remained fused with LAP-heavy chain (LAP-HC) due to incomplete cleavage on furin-2A sequence in the cells. HC: heavy chain. S: serum alone. M: Protein marker.

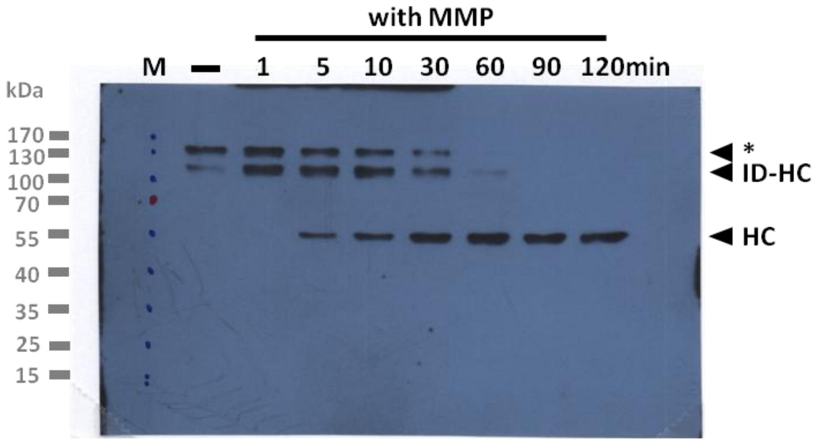

Figure S8: The original blot for cropped blot presented in Figure 6A.
Asterisks (*) indicate the light chain still fused with inhibitory domain-heavy chain (ID-HC) due to incomplete cleavage on furin-2A sequence in the cells. In the Figure 6A, contrast of original blot was enhanced to more clearly visualize the weaker protein bands. HC: heavy chain. with MMP: with MMP-2 incubation. M: Protein marker.

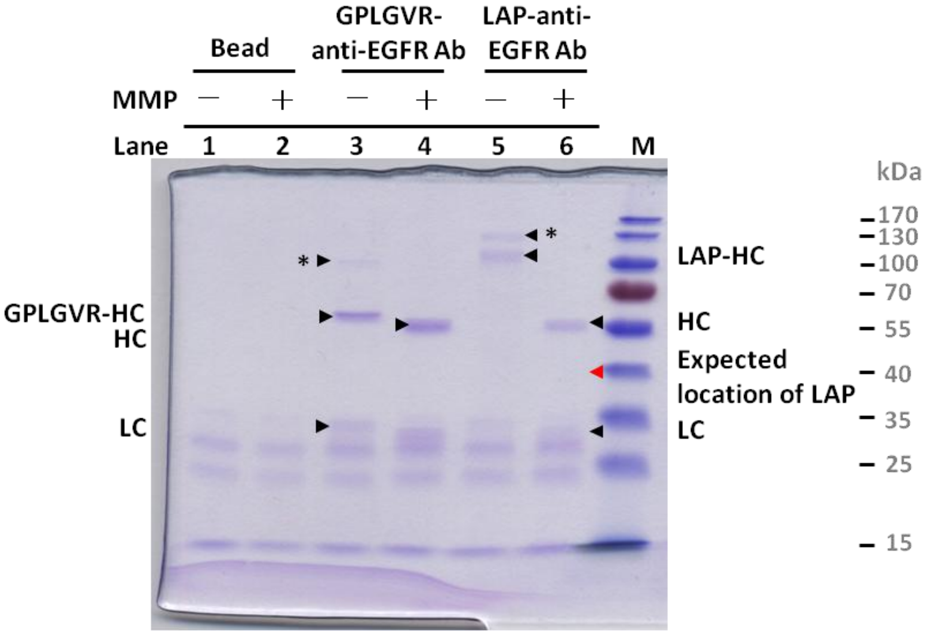

Figure S9: The original gel for cropped gel presented in Supplementary Figure S2.
In the Supplementary Figure S2, image contrast was enhanced to more clearly visualize the weaker protein bands. Asterisks (*) indicate the light chain (LC) still fused with heavy chain (HC) or LAP-heavy chain (LAP-HC) due to incomplete cleavage on furin-2A sequence in the cells. The location of LAP domain in the gel is indicated by a red arrowhead. No ~40 kDa (the molecular weight of LAP) protein band was noted in MMP-2 treated LAP-anti-EGFR antibodies in the gel (lane 6). GPLGVR-HC: MMP-2 substrate peptide-linked heavy chain. Bead: protein A sepharose beads alone. M: prestained protein marker.

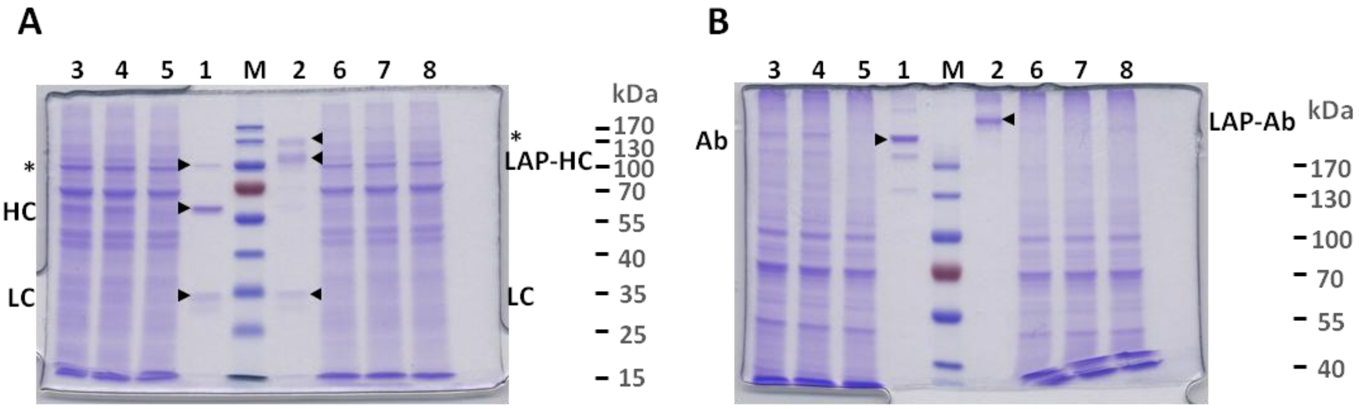

Figure S10: The original gels for cropped gels presented in Supplementary Figure S1 and S4. In Supplementary Figure S4, image contrast was enhanced to more clearly visualize the weaker protein bands. (A) The original gel for panel A under reducing condition presented in supplementary Figure S4A. (B) The original gel for panel B under non-reducing condition presented in supplementary Figure S4B. The lane 1 and lane 2 from panel B was also the original gel presented in Supplementary Figure S1A and S1B, respectively. Lane 1: purified anti-EGFR antibodies; Lane 2: purified LAP-anti-EGFR antibodies; Lane 3 and 8: culture supernatant of antibody-producing Expi293 transfectants; Lane 4 and 7: flow-through fraction from G-25 resins; Lane 5 and 6: flow-through fraction from protein A beads. M: prestained protein marker. Under reducing conditions, asterisks (*) indicate the light chain (LC) was remained fused with heavy chain (HC) or LAP-heavy chain (LAP-HC) due to incomplete cleavage on furin-2A sequence in the cells. Under non-reducing conditions, the full anti-EGFR antibodies and LAP-anti-EGFR antibodies are indicated by arrowheads.
